# The Role of Bacterial Membrane Vesicles in the Dissemination of Antibiotic Resistance and as Promising Carriers for Therapeutic Agent Delivery

**DOI:** 10.3390/microorganisms8050670

**Published:** 2020-05-05

**Authors:** Md Jalal Uddin, Jirapat Dawan, Gibeom Jeon, Tao Yu, Xinlong He, Juhee Ahn

**Affiliations:** 1 Department of Medical Biomaterials Engineering, College of Biomedical Science, Kangwon National University, Chuncheon, Gangwon 24341, Korea; jalalmbe@kangwon.ac.kr (M.J.U.); Jirapat@kangwon.ac.kr (J.D.); ss01208@kangwon.ac.kr (G.J.); 2 Shandong Institute of Parasitic Diseases, Shandong First Medical University & Shandong Academy of Medical Sciences, Jining 272033, China; yutao5816@126.com; 3 Institute of Translational Medicine, Medical College, Yangzhou University, Yangzhou 225001, China

**Keywords:** outer membrane vesicle, antibiotic resistance, virulence, vaccine, therapeutic agent delivery

## Abstract

The rapid emergence and spread of antibiotic-resistant bacteria continues to be an issue difficult to deal with, especially in the clinical, animal husbandry, and food fields. The occurrence of multidrug-resistant bacteria renders treatment with antibiotics ineffective. Therefore, the development of new therapeutic methods is a worthwhile research endeavor in treating infections caused by antibiotic-resistant bacteria. Recently, bacterial membrane vesicles (BMVs) have been investigated as a possible approach to drug delivery and vaccine development. The BMVs are released by both pathogenic and non-pathogenic Gram-positive and Gram-negative bacteria, containing various components originating from the cytoplasm and the cell envelope. The BMVs are able to transform bacteria with genes that encode enzymes such as proteases, glycosidases, and peptidases, resulting in the enhanced antibiotic resistance in bacteria. The BMVs can increase the resistance of bacteria to antibiotics. However, the biogenesis and functions of BMVs are not fully understood in association with the bacterial pathogenesis. Therefore, this review aims to discuss BMV-associated antibiotic resistance and BMV-based therapeutic interventions.

## 1. Introduction

Over the last few decades, antibiotic resistance in bacteria has been a serious global threat to public health [1]. Antibiotic-resistant bacteria can survive current antibiotic regimens, resulting in frequent therapeutic failure [2]. The emergence of antibiotic-resistant bacteria spurred the necessity of developing new antibiotics [3,4]. The fundamental understanding of antibiotic resistance mechanisms is an important step in the development of effective therapeutic regimens. The intracellular levels of antibiotics are synergistically regulated by efflux pump systems and membrane permeability barriers [3]. Recently, it has been recognized that bacterial membrane vesicles (BMVs) may play a role in antibiotic resistance. Therefore, understanding the roles of BMVs can provide directions for the control of antibiotic-resistant pathogens.

The structural features of the bacterial outer membrane play an important role in the rapid adaptation to environmental stresses such as cold, heat, and antibiotic treatments, resulting in the evolution of antibiotic resistance in bacteria [2,5]. Therefore, the structure, biogenesis, function, and regulation of BMVs could be a new research area in connection with antibiotic resistance [6]. Although the biological functions of BMV-containing components have been considered important for understanding the mechanisms related to antibiotic resistance, there is still a lack of information on the biogenesis of BMVs in terms of antibiotic resistance [7]. Therefore, this review addresses the possible roles of BMVs in the control and prevention of the emergence of antibiotic-resistant bacteria.

## 2. Terminology and Characteristics of Bacterial Membrane Vesicles

**Figure 1:**
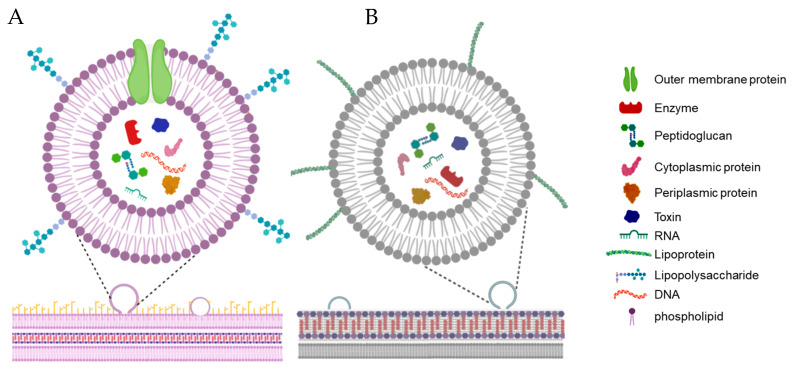
Structural characteristics of Gram-negative (**A**) and Gram-positive (**B**) bacterial membrane vesicles.

The term BMV has been used to describe various extracellular substances, known as outer membrane vesicles (OMVs), which are specifically released from Gram-negative bacteria. Similarly, Gram-positive bacteria and archaea produce vesicles, known as membrane vesicles (MVs), and eukaryotic bacteria secrete surface and cellular lipids and proteins, named exosomes or microvesicles [8,9,10]. Therefore, the term OMV is not inclusive as there are many vesicle-producing Gram-positive bacteria. The BMV could be an inclusive term for membrane vesicles released from both Gram-negative (BMV_GN_) and Gram-positive (BMV_GP_) bacteria. The BMVs are nano-sized spherical membrane particles released from the bacterial membranes, encapsulating proteins, toxins, peptidoglycan, lipopolysaccharides (LPSs), and nucleic acids [11]. The BMVs have less than 370 kbp in DNA and are 10–300 nm in diameter [6,12]. The BMVs play an important role in bacterial cell-to-cell interactions [13]. The structural characteristics of BMVs (Figure 1) contribute to bacterial resistance to different types of environmental stresses [2,5].

## 3. Isolation and Purification of Bacterial Membrane Vesicles

Isolation, purification, and storage techniques have been developed to collect BMVs, which are essential steps for understanding structural and functional characteristics of BMVs [14]. Those techniques include conventional gradient centrifugation, column chromatography, immune affinity-based separation, and the proteomic approach [14,15,16]. The amount and content of BMVs varies depending on the bacterial growth conditions and genetic variation [17]. High purity is essential to characterize BMVs and applies for delivery system and vaccine development [18]. Differential centrifugation is used to remove non-BMVs in bacteria by serial centrifugation from 300 to 2000× *g*, and 10,000 to 100,000× *g* [19]. However, the differential centrifugation technique provides low yield and insufficient purity due to the repetitive ultracentrifugation [20]. Density gradient ultracentrifugation is applied to increase the separation efficiency of BMV particles according to the unique buoyant densities [21]. In addition, this method increases the yield of BMVs in terms of the purity of BMV fraction and the quantity of BMV proteins and RNAs. Hence, the density gradient ultracentrifugation method is considered one of the most suitable ways to purify BMVs [22]. However, the substantial loss of BMVs occurs in this method due to its complex, strenuous, and time consuming (>2 days) nature as well as its requirement for expensive equipment [23].

The filtration method is used to purify BMVs according to size. Many membrane filters with various pore sizes are useful for separating BMV particles. Ultrafiltration is a tangential flow filtration method with membrane pore sizes between 0.001 and 0.1 µm. The ultrafiltration can remove high molecular-weight substances such as viruses and organic and inorganic polymeric molecules [24]. However, this method is unable to efficiently purify the BMV fraction from non-BMV contents [25]. Gel filtration is known as size exclusion chromatography. This method can isolate molecules that have a different hydrodynamic radius and isolate proteins, polysaccharides, and BMVs. However, this method has a disadvantage, which is that it requires pre-processing, such as via ultracentrifuge or ultrafiltration [26,27]. Precipitation is usually used to purify proteins. Proteins are aggregated by adding a high concentration of salts, which can disturb the surface charges and hydrogen bonds to be easily isolated by centrifugation. This technique can also be used to isolate BMVs through dialysis [28].

A two-phase system with polyethylene glycol (PEG) and dextran is used to increase the purity of BMVs [29]. The BMVs and proteins are preferentially accumulated in the dextran phase and PEG phase, respectively. The repeated replacement of PEG can improve the purity of BMVs in the cell mixtures [30]. The surface components of BMVs, including proteins, lipids, and polysaccharides, are potential ligands binding to receptors. The specific binding affinity between ligands and receptors can be used to purify BMVs [31]. The affinity-based methods can improve the purity and selectivity of BMVs, but have disadvantages such as expensive antibodies, low isolation efficacy, and limited sample volume [32]. Thus, the affinity-based methods are further improved using a His-tag mutant and immobilized metal affinity chromatography (IMAC) [33]. The His-tag technology coupled with IMAC can selectively purify BMVs. The plasmid-encoded transmembrane proteins provide a His-tag sequence for bacterial outer membranes. Microfluidic devices based on microelectronic technology can adjust fluidic movement, and are able to handle viscous media in volumes ranging from picoliters to microliters. Microfluidic devices can reduce the sample quantity and processing time [31]. A microfluidic device with an immunoaffinity and membrane filter can rapidly and efficiently purify BMVs [34,35]. Since the purification methods for BMVs have advantages and disadvantages, an improved method still is needed to isolate BMVs with high purity.

## 4. Biogenesis of Bacterial Membrane Vesicles

The biogenesis (vesiculation) of BMVs is a physiological process, but its mechanisms still remain unknown [36]. BMVs might be produced through stochastic or regulated biogenesis mechanisms [37]. Current hypotheses on vesiculation propose that BMVs are forced out of the cell through the cell membrane and/or cell wall and contain the enzymes to destroy the peptidoglycan [10,38,39,40]. The vesiculation results from the outcome of the normal turnover of bacterial cells [41]. The BMVs are independently released from the bacterial cell envelope without alteration in membrane integrity [42]. The production of BMVs is an important step for bacteria to adapt to various stresses, including antibiotic treatment, heat, and acid [43]. The BMVs are constitutively produced in Gram-negative bacteria [5]. The factors which affect the BMV secretion in Gram-negative bacteria include various physiological and environmental stresses [44]. For instance, BMV production is triggered by antibiotics, high temperature, oxidizing agents, and nutrients [45]. In addition, two-component regulatory systems, such as PhoP/Q and PmrA/B, can modify LPS structure and regulate outer membrane proteins (OMPs) under acidic conditions [5]. *Pseudomonas* quinolone signals (PQSs), produced and secreted by the *Pseudomonas* species, can contribute to the generation of BMVs. The release of BMVs is attributed to the cell membrane charge and perturbation, including the interactions of LPS with divalent ions and membrane disruption stimulated by antibiotics, chelators, or hydrophobic compounds [8,46]. The BMV production is decreased in the presence of divalent ions (Mg^2+^) [8]. The production of BMVs from *Staphylococcus aureus*, *Bacillus subtilis*, and *Streptococcus mutans* occurs during coagulation and biofilm formation. The bacterial growth phases also contribute to the changes in the size and amount of BMVs; small, medium, and large BMVs are produced, respectively, in the early log phase, stationary phase, and mid-log phase [8,47]. Many researchers have made efforts to understand the regulation of BMV formation at the genetic level. The mutations in genes *ypjA* and *nlpA*, encoding cell envelope-localized proteins, can cause a decrease in the crosslinking level in peptidoglycan synthesis and promote the production of BMVs [48]. The overexpression of the genes associated with envelope stress response-related proteins can increase the production of BMVs without changes in membrane integrity [42]. Furthermore, the σ^E^ pathway could be activated in response to the misfolded OMP by upregulating several genes encoding periplasmic chaperones and proteases [49,50]. This could be due to specific σ^E^- regulated proteins [50]. The BMV-associated RNAs can regulate the formation of vesicles. A previous study demonstrated that the small RNA in *Vibrio cholerae*, VrrA, can block the expression of OmpA, which stabilizes the outer membrane and peptidoglycan cross-links of the bacterial envelope and the suppression of OmpA, leading to the increase in vesiculation [51]. Moreover, the sRNAs, MicA, and RseX in *Escherichia coli*, and MicA and RybB in *Salmonella* have also been reported to downregulate the OmpA [52,53].

The BMV-producing bacteria induce an envelope stress response that provides the benefits of adaptation in the bacterial community [37]. The production of BMVs can be stimulated by envelope stress and other environmental conditions [42]. Moreover, the membrane-associated vesicular proteins, such as outer membrane proteins (OMPs) and transport proteins, act as functional barriers for various substances in accordance with hydrophobicity, electric charge, and polarity, leading to the development of antibiotic resistance in bacteria [54,55]. The decreased permeability of outer membranes results in the increased resistance to antibiotics such as colistin and polymyxin B [56]. Successively, the antibiotic-resistant bacteria are involved in the production of BMVs containing antibiotics [1]. In addition, antibiotics, including gentamicin, polymyxin, D-cycloserine, and mitomycin C, can induce the production of BMVs from *Pseudomonas* and *Shigella* [57,58]. A similar observation has also been reported for the production of BMVs in *Escherichia coli* O104:H4, and O157:H7 was increased in the presence of antibiotics such as ciprofloxacin, meropenem, fosfomycin, and polymyxin B [59]. The secreted BMVs help bacteria to survive antibiotic treatment by acting themselves as targets for antibiotics (Table 1) [60]. Interestingly, the BMVs bind peptide antibiotics with high affinity but do not bind well to hydrophobic antibiotics [1]. *Mycobacterium* BMVs contain various proteins, including virulence-associated proteins and toll-like receptor (TLR) ligands [61].

## 5. Biological Functions of Bacterial Membrane Vesicles

The BMVs play an important role in bacterial survival associated with intracellular communication under environmental stress conditions. The BMVs produced by Gram-negative bacteria contain lipids, proteins, LPSs, and genetic materials [13]. The vesiculation is influenced by the lipid A deacylase (PagL) [76]. The BMVs contain glycerophospholipids, phosphatidylglycerol, phosphatidylethanolamine, and cardiolipin in enterotoxigenic *E*. *coli*; phosphatidylglycerol and phosphatidylethanolamine in *Pseudomonas syringae*; and phosphatidylglycerols in *Pseudomonas aeruginosa* [77]. In addition, the enzymes that hydrolyze β-lactam antibiotics are packaged inside the BMVs of *P*. *aeruginosa* and then released by the cell [78]. The BMVs of *Yersinia pestis* contain a penicillin-binding protein activator that regulates peptidoglycan synthesis [79]. The BMVs produced by Gram-positive bacteria contain enzymes, toxins, hemolysin, and IgG-binding proteins. The BMVs have multifunctional properties that play a role in colonization, survival, antibiotic resistance, immunomodulation, autolysins, biofilm formation, virulence, and pathogenesis [41,80,81,82]. 

The BMVs in *E*. *coli* act as carriers to remove misfolded proteins from the bacterial cells [42]. The components of BMVs released from Gram-positive bacteria differ from those of BMVs released from Gram-negative bacteria that contain LPS and periplasmic components [38]. The misfolded proteins are accumulated in the periplasmic space and prevented by chaperones and proteases (DegP) [83]. The virulence factors, including β-lactamase, hemolysins, phospholipases, lipases, ureases, chitinases, proteases, molecular chaperones, and toxins, are found in BMVs [44,47,84]. For example, the BMVs in *P. aeruginosa* contain virulence factors such as proteases and hemolysin, which disrupt the quorum-sensing molecules and can lead to the lysis of Gram-negative and Gram-positive bacteria [84]. Previous studies have demonstrated that the BMVs contain various components, including periplasmic and cytoplasmic components, the inner membrane, and OMPs [6,43,47]. Therefore, the OMPs in BMVs, such as OmpA, OmpC, and OmpF, can act as virulence factors for evading the host immune response [13,55,85]. The BMVs provide many benefits for bacteria, including protection against enzymatic degradation, target specificity, sustainable toxin delivery, antibiotic resistance, immune evasion, bacterial invasion, and adherence [36,43,46,47]. The BMVs can protect bacteria from hydrophobic and peptide antibiotics that enhance membrane affinity [1,67]. Additionally, they help bacteria to increase their resistance to colistin and β-lactams, but do not cause any changes in the susceptibility to ciprofloxacin, streptomycin, and tetracycline [67]. The vesicles secreted from Gram-positive and Gram-negative bacteria can be possibly used for therapeutic development and antigen display [86].

## 6. Gene Transfer Potential of Bacterial Membrane Vesicles

The BMVs carry genetic materials and virulence factors, which are responsible for antibiotic resistance and pathogenesis. Pathogenic bacteria are more likely to secrete BMVs than nonpathogenic bacteria in order to survive under stressful conditions through biofilm formation and gene/nutrient transfer [15]. Various genetic materials have been identified from the BMVs produced by Gram-negative and Gram-positive bacteria (Table 2) [12,87,88]. Many studies have found the presence of DNA in BMVs that can be originated from chromosomes, plasmids, and bacteriophages [17,89]. Several types of RNAs, such as mRNA, rRNA, sRNA, and tRNA, have also been identified in BMVs [90]. Recent studies have reported that the BMVs produced by *Neisseria gonorrhoeae*, *Prochlorococcus* sp., and *Porphyromonas gingivalis* contain both DNAs and RNAs [91,92]. DNAs are supposed to be trapped into BMVs by several ways: by means of a cytoplasmic route, where the DNA from the cytoplasm is trapped with other components in inner and outer membrane vesicles; through a periplasmic route, where the DNA from the cytoplasmic site relocates to the periplasmic space, followed by arrest in BMVs; by an extracellular route, probably because of broken BMVs that re-annealed after liberation from the bacteria; or due to cell death [40,89,93,94]. In addition, bacteriophages can directly inject their DNA into BMVs [91]. RNAs together with the ribosomal proteins are encapsulated into BMVs through the routes described for DNA [92,95].

The BMVs can act as a vehicle for horizontal gene transfer into bacteria cells [105]. The gene transfer via BMVs is responsible for the microbial fitness determinants, including antimicrobial resistance, metabolic property, and virulence [1,89,102]. The antibiotic-sensitive *E*. *coli* can survive due to the BMV-containing β-lactamases responsible for the resistance to ampicillin, cefoperazone, and cefotaxime. Furthermore, the antibiotic resistance genes can be transferable to other bacteria through BMVs. For example, the BMV-producing *E. coli* contain transferable colistin and melittin resistance genes to *P. aeruginosa* and *A. radioresistens*, which lead to acquired resistance to membrane-disrupting antibiotics colistin and melittin [67]. Likewise, the BMVs from *Acinetobacter baumannii* are capable of transferring the OXA-24 carbapenemase gene, leading to the dissemination of antibiotic resistance in bacteria [89]. Additionally, the antibiotic resistance in *E. coli* is increased in the presence of BMVs. This assumes that the β-lactamases could be packaged into the vesicles during the biogenesis of BMVs due to their location in the periplasmic site of bacteria [106]. BMVs from *Pseudomonas aeruginosa* have been found to carry chromosomal β-lactamases, which can be transferred to other bacteria [72]. Furthermore, the cephalosporinase gene-containing BMVs secreted from *Bacteroides* spp. help gut pathogens exposed to β-lactam antibiotics survive [107]. Gram-positive bacteria, such as *S*. *aureus*, also produce BMVs containing the *blaZ* gene responsible for ampicillin resistance [108]. Multidrug resistant (MDR) bacteria acquire antibiotic resistance through many different mechanisms, including efflux pump activity, membrane permeability, biofilm formation, and enzymatic inactivation [106]. Bacterial porins and efflux pumps on the outer membrane play an important role in the development of multidrug resistance by selectively uptaking substrates and expelling intracellular antibiotics [41,105,109]. In addition, the BMVs involve interspecific and intraspecific transport of virulence genes. The BMV-producing bacteria contain multiple virulence factors, including proteases and leukotoxin from *Actinobacillus actinomycetemcomitans*, shiga toxin from *E. coli*, the *vacA* gene from *Helicobacter pylori*, and β-lactamase and alkaline phosphatase from *P*. *aeruginosa* [89]. Similarly, *Bacillus anthracis* produces BMVs containing toxins and anthrolysin, which can be transported to the host cells [110]. Therefore, BMVs could act as a vector in horizontal gene transfer that plays a vital role in the dissemination of antibiotic resistance among the bacteria. The BMVs can stimulate the formation of biofilm, and BMVs within biofilm can inactivate harmful molecules such as antibiotics, complements, and antibodies [13,111]. Quorum sensing (QS) is the strategy for surviving in a high density of bacteria, which produce quorum sensing molecules, known as auto-inducers, involved in adherence and biofilm formation. A previous study has reported that the hydrophobic QS molecules packed in BMVs are released from *Vibrio harveyi* during the stationary phase [112]. Moreover, the BMVs can facilitate the trafficking of QS signaling molecules produced by *P*. *aeruginosa* [41]. 

## 7. Proteomic Properties of Bacterial Membrane Vesicles

**Figure 2:**
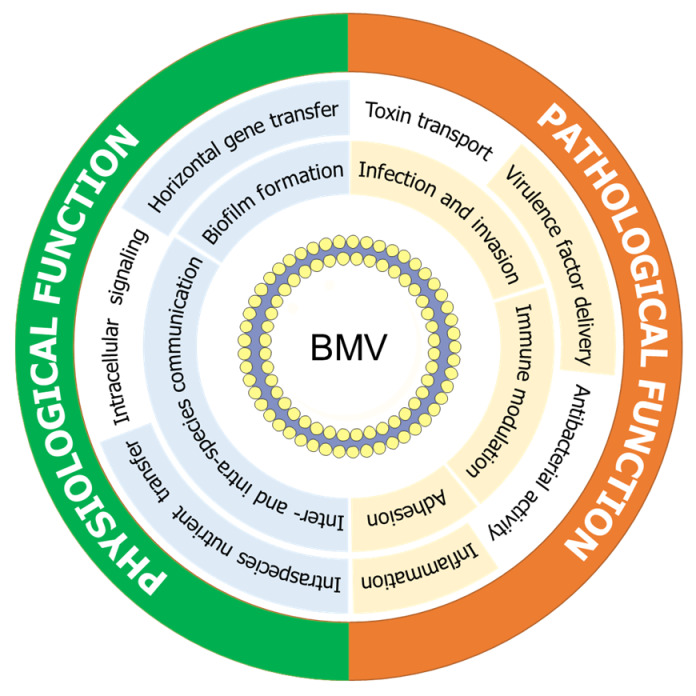
Physiological and pathological functions of bacterial membrane vesicles.

Proteins mostly contribute to the functional property of bacterial BMVs. Many researchers have extensively studied the identification of BMV-containing proteins using MS-based high-throughput proteomic analysis [113]. The conserved vesicular proteins can also provide valuable information for the biogenesis of BMVs in Gram-negative and Gram-positive bacteria [114]. The BMVs carry DNAs, and RNAs, and the translation of outer membrane proteins might coincide with their integration into the membrane, resulting in transcriptional and ribosomal proteins being integrated into BMVs [12,102]. The vesicular proteins OMPs, Tol-Pal, YbgF, and Lpps are involved in outer membrane integrity, which can contribute to the production of BMVs from the bacterial cell surface [115]. The peptidoglycan fragments are degraded by murein hydrolases, MltA, MipA, MltE, and SLP, and accumulated in the periplasmic site, resulting in the release of BMVs [116]. The cell wall-modifying enzymes in Gram-positive bacteria, including penicillin-binding proteins, lipoteichoic acid synthase, and *N*-acetylmuramoyl-*l*-alanine amidase, act as peptidoglycan hydrolase, leading to the vesicle formation [117]. The vesicular proteins are involved in a wide range of physiological and pathological functions, including host cell adhesion and invasion, antibiotic resistance, host cell destruction, immune system modulation, biofilm formation, and virulence promotion (Figure 2) [47]. 

The proteins secreted from BMVs have several distinct advantages over general secretory pathways, which are inaccessible to extracellular enzymes and transportable for a long distance [118]. For example, the vesicular proteins Ata, BabA, SabA, and OmpA, derived from *H*. *pylori* and *A*. *baumannii*, mediate adhesion to host cells [119,120]. The vesicular Ail protein can enhance the invasiveness of *E. coli* [121]. Furthermore, Staphopain A, a protein produced from *S. aureus* BMVs, plays an essential role in cellular invasion [122]. The BMVs produced by Gram-negative and Gram-positive bacteria can carry β-lactamases (AmpC and BlaZ), resulting in enhanced antibiotic resistance to β-lactam antibiotics [66,78]. The BMVs secreted from *S. aureus* are enriched in penicillin-binding proteins, which usually bind to β-lactam antibiotics and contribute to methicillin resistance [123]. The BMVs also harbor many multidrug efflux pump-related proteins (Mtr, Mex, and TolC) [1,124]. In addition, the BMVs carry several virulence factors, including toxins (α-hemolysis, cytolysin A, heat-labile enterotoxin, leukotoxin, shiga toxin, Cif, and β2 toxin), digestive enzymes (alkaline phosphatase, elastase, and haemolytic phospholipase C), and superantigens (SEQ, SSaA1, and SSaA2), which can play roles in damage to host cells and modulate the host immune responses [13,123,125,126,127,128,129]. The murein hydrolases (MltA and SLT), endopeptidase L5, peptidoglycan hydrolase, and amidase in BMVs are involved in killing competing bacteria by cell wall degradation [117,130,131]. The ATP-binding cassette (ABC) transporters for specific nutrients (BtuB, FhuA, and FadL) and hemin-binding protein C in BMVs have been reported to be nutrient sensors and carriers, responsible for the bacterial survival in nutrient deficiency [132,133]. The *Porphyromonas gingivalis* BMVs contain heme-binding lipoprotein (HmuY), which might be helpful in biofilm formation and cell survival during starvation periods [134]. The pathogen-associated lipoproteins from BMVs can promote inflammatory responses in the host [135]. Moreover, the BMVs secreted from *Mycobacterium tuberculosis* and *Mycobacterium bovis* contain lipoproteins, including LpqH, LppX, LprA, and PstS1, that act as virulence factors [136]. Taken together, the vesicular proteins can play significant roles in biogenesis and pathogenesis (Table 3).

## 8. Bacterial Membrane Vesicle-Based Therapeutic Approaches

The effectiveness of antibiotics in treating infectious diseases has been challenged due to the rapid spread of multidrug-resistant bacteria [163]. Therefore, alternative therapeutic methods are desperately needed in the clinical field. BMVs are nano-sized-vectors, responsible for the spread of virulence factors such as bacterial antigens, toxins, and antibiotic resistance-related genes [1]. Because of their structural and functional characteristics, the BMVs can be used to develop drug delivery platforms that prevent enzymatic degradation [164] and evade immune-mediated elimination [165]. The BMVs are promising candidates for developing antibiotic carriers and vaccines [14,86,165]. The BMVs contain pathogen-associated molecular patterns (PAMPs), which play an important role in innate immune stimulation and adaptive immune responses [43]. Bioengineered BMVs also have great benefits, including high specificity, loading efficacy, and stability [81]. Gentamicin-induced vesicles contain gentamicin, which can be used for the production of antibiotic carriers [153]. A recent study has observed that biocompatible BMVs encapsulate antibiotics and small interfering RNAs without adverse side effects [165]. In addition, BMVs can also encapsulate target antigens into the vesicle cavity or mosaic on the outer membrane through a certain mechanism, which is recognized by the host cell and causes an immune response, known as antigen presentation. BMVs also contain a variety of antigens, in addition to Toll-like receptor (TLR) agonists with natural adjuvant effects, including OMPs, lipoproteins, and LPSs. The advantages of BMVs include that they easily enter through the tissue cells and their surface molecules can be recognized by the immune system. Furthermore, the antigen-presenting dendritic cells can be stimulated by BMVs, leading to the induction of T and B cell-mediated immune protection [166]. Therefore, the application of BMVs has a very promising future as vaccine delivery vectors and in recombinant multivalent vaccines. For instance, *E. coli* BMVs can integrate and present heterologous OMPs and periplasmic proteins, and also can express *Yersinia enterocolitica* Ail protein with adhesion and invasion functions [121]. The modified BMVs of the *Salmonella* Typhimurium vaccine were used to present the *Streptococcus pneumoniae* model antigen, PspA, in the vesicle cavity, and provide immunization with nasal drops in mice [167]. The specific IgA antibody against PspA protects mice from lethal *S. pneumoniae*. Schroeder et al. [168] used proteins that penetrated the outer membrane, periplasmic space, non-adhesion bacterial surface protein, and KMP-11 antigen of the *Leishmania* parasite to fuse and express on *Salmonella* BMVs. Compared with the direct presentation of the KMP-11 antigen by attenuated *Salmonella*, its immune-boosting effect was increased by 40 times. A BMV delivery system was successfully established by fusion expression of heterologous antigens and OmpA genes, which provides a theoretical basis for BMVs as vaccine vectors [169]. Chen et al. [170] used *E. coli* BMVs to express a fusion protein and bacterial hemolysin CyA protein to induce an immune response against green fluorescent protein. Previous studies have shown that presenting heterologous antigens on the surface of BMVs can induce an effective immune response [171]. The BMVs containing immune-related molecules are a potential tool for vaccine development due to their immunogenicity and adjuvanticity [36,43,68,80,172]. The BMVs containing β-lactamase protein (BlaZ) are released from Gram-positive bacterium *S*. *aureus*. The development of an anti-β-lactamase antibody from BMVs can be used to increase the susceptibility of β-lactamase-producing bacteria to β-lactam antibiotics [1,173]. 

The BMVs released from pathogenic bacteria contain various cell surface components, such as capsular polysaccharides (CPSs) and LPSs, which can be specific targets for vaccine development [174]. Vaccines are considered to be the most direct and effective strategy to deal with bacterial diseases in the post-antibiotic era [175]. The membrane components contained in BMVs can stimulate the host to produce adaptive immune memory. The LPS contained in BMVs as an adjuvant can be used for a non-replicating vaccine. Since BMVs have achieved good results as a vaccine to prevent *N*. *meningitidis* infections, researchers have continued exploring the role of BMV vaccines against other pathogenic bacteria. The BMVs are naturally released by bacteria into the surrounding environment under normal growth conditions, which contain outer membrane antigens with natural conformation. Previous studies have shown that the components containing *P*. *aeruginosa* BMVs induced strong inflammatory responses [135]. The nasal immunization of mice with *Hemophilus influenza* BMVs not only induced strong mucosal and humoral immune responses, but also protected mice from heterologous influenza *Haemophilus* infections [176]. In addition, a mixture of *Pasteurella multocida* and *Mannheimia haemolytica* BMVs could induce strong specific mucosal and humoral immune responses [177]. These findings suggest that multiple BMV vaccines can be developed to protect against diseases caused by heterogeneous bacterial infections. Petersen et al. [178] immunized a cynomolgus monkey with *Burkholderia pseudomallei* BMVs, and the BMVs provided humoral immune protection against related proteins and LPSs.

BMVs contain many immunogens, including pathogenicity island-encoded proteins, OMPs, and chaperones. The composition of OMPs modulated by stresses and sRNA is responsible for the biogenesis of BMVs [179]. MicA induces OmpA and OmpC, which are involved in BMV production and immune response against bacteria [179]. The expression of OmpA, which is regulated by small RNAs, is negatively associated with the production of BMVs in *Salmonella* (RybB), *Vibrio* (VrrA), and *E*. *coli* (MicA) [37]. The various components of BMVs can be used to develop multivalent immunogenic vaccines [43,180]. For instance, the factor H-binding protein in *N*. *meningitides* plays an important role as a vaccine candidate. Additionally, the antigens and immune stimulators extracted from BMVs can be used for vaccine development. Previous studies have demonstrated that the *Mycobacterium* BMVs containing vesicle-associated antigens can be used for vaccine development to treat tuberculosis and potential biomarkers to selectively detect antibiotic-resistant bacteria [61,181]. Adjuvants are commonly used to combine with antigens to increase a weak immune response system [182]. Furthermore, the aluminum adjuvant was first practically applied for a human vaccine that was proven to be safe according to the vaccination schedules [183]. The benefits of using adjuvants include low cost, widespread circulation, and effective immune stimulation [184].

Adjuvants are substances that can assist vaccines by enhancing antigen-specific immune responses. The immune response induced by nasal immunity is not sufficient, so protein vaccines such as cholera toxin and *E*. *coli* heat labile enterotoxin may be used as adjuvants to increase the immune response [185]. However, the vaccine adjuvants have a disadvantage regarding safety. For example, nasal influenza vaccine mixed with *E. coli* heat-resistant toxins as a mucosal adjuvant may cause facial paralysis [186]. BMVs act as relatively safe adjuvants and can induce a highly effective immune response. *N*. *meningitidis* BMV vaccine has been used in many countries and can provide effective immune protection to adults or children [187]. Mixed inoculation of *N*. *meningitidis* BMVs with an influenza vaccine can significantly enhance the mucosal and systemic immune response [188]. In addition, several studies have found that mixed immunization of mice with BMVs and tumor-associated antigen gangliosides with low immunogenicity can stimulate the immune response to tumor antigens, enhancing the ability to resist cancer invasion [189]. Previous reports have proved that the immunopotent combination of virus-like particles (VLPs) and BMVs of *N. meningitidis* group B could induce anti-HIV-1 IgG and IgG2a, and also increase the production of IFN-gamma [184]. BMVs are also an effective mucosal adjuvant. Sardinas et al. [190] immunized mice by mixing OMVs of *N*. *lactis* with hepatitis B surface antigen HBsAg. Compared with the control group immunized with the hepatitis B surface antigen HBsAg alone, the mixed group induced high levels of HBsAg-specific IgA and IgG antibodies. Although a large number of tests have shown that BMVs can be a good vaccine choice, BMVs without any modification still have toxicity as a vaccine. For Gram-negative bacteria, BMV vaccination is limited by the incorporation of LPS or lipooligosaccharide (LOS) into the bilayer of BMVs. In order to use BMVs as a safe delivery vector, Kim et al. [169] mutated the MsbB gene encoding *E. coli* lipid A acyltransferase, which reduces the toxicity of LPS. BMVs derived from *S. aureus* could contain some species-specific virulence factors responsible for the safety of a potential vaccine [191]. However, Yuan et al. constructed an *agr* locus deletion mutant of the *S. aureus* strain (RN4220-Delta*agr*) to reduce potential toxicity. Administration of such engineered (Delta*agr*) BMVs in mice induced antibodies against all four dengue virus serotypes [192]. In addition, probiotic bacteria are known as generally recognized as safe (GRAS). BMVs from probiotic Nissle 1917 and gut resident *E*. *coli* strains distinctly modulate human dendritic cells and subsequent T cell responses [193]. *Lactobacillus plantarum*-derived BMVs can effectively protect atopic dermatitis induced by *S. aureus*-derived BMVs [194]. Therefore, the expression and further encapsulation of proteins into BMVs could represent a scientific novelty in BMV vaccination.

## 9. Conclusions

BMVs, derived from Gram-negative and Gram-positive bacteria, are considered to play a crucial role in intercellular communication between bacteria, and between bacteria and host. However, the mechanism of BMV biogenesis and its interaction with the host are still far from our understanding. Bacteria tend to produce more BMVs as a survival mechanism in response to unfavorable conditions such as antibiotic exposure. BMVs play an important role as carriers of antibiotic-related proteins and in inactivating antibiotic enzymes. Therefore, these vesicles are the major protective agents for bacterial growth and survival in the presence of antibiotics. In addition, purification and production are potentially important for BMVs to be used as vaccines. Vaccines have been widely applied to protect human health from infectious diseases. Recently, BMVs have gained attention as potential vaccine candidates due to their stability and protection against pathogens. BMVs have been applied as vaccines for inducing protective immune responses to human pathogens such as *N. meningitides*, *Bordetella pertussis*, and *B. pseudomallei*. One promising vaccine against pathogenic bacteria is the cell surface polysaccharide, coordinated with BMV formation. BMVs can be a promising platform for vaccine development. Therefore, BMVs have great potential for the design of a vaccine delivery platform to effectively control antibiotic-resistant pathogens.

## Figures and Tables

**Table 1:** Specific components of bacterial membrane vesicles (BMVs) as targets for antibiotics.

Bacterium	Receptor	Ligand	Reference
*Staphylococcus aureus; Enterococcus faecalis; Streptococcus* spp.	Monomeric membrane phospholipids	Daptomycin, nisin, pexiganan, melittin	[62,63]
*Escherichia coli*	Lipid and protein	Polymyxin B and E	[64,65]
*Moraxella catarrhalis; Escherichia coli*	Hydrolytic enzymes	Amoxicillin, cefaclor Melittin, penicillin, methicillin	[66] [67,68]
*Burkholderia cenocepacia*	Hydrophobic lipocalins	Rifampicin, norfloxacin, ceftazidime, polymyxin B	[60]
*Pseudomonas aeruginosa; Streptococcus pneumoniae; Klebsiella pneumoniae*	Capsular polysaccharides	Polymyxin B	[69,70,71,72,73]
*Pseudomonas aeruginosa; Staphylococcus epidermidis; Haemophilus influenzae*	eDNA	Kanamycin, tobramycin, vancomycin, human β-defensin-3, gentamicin, amikacin	[70,74,75]

**Table 2:** Genetic materials in bacterial membrane vesicles (BMVs).

Genetic Material	Species	Reference
Chromosomal DNA	*Escherichia coli*	[91]
	*Clostridium perfringens*	[96]
	*Neisseria gonorrhoeae*	[12]
	*Porphyromonas gingivalis*	[97]
	*Prochlorococcus* sp.	[98]
	*Ruminococcus* spp.	[87]
	*Shewanella vesiculosa*	[99]
	*Mycobacterium tuberculosis*	[100]
	*Streptococcus mutans*	[101]
Plasmid DNA	*Acinetobacter baumanni*	[94]
	*Acinetobacter baylyi*	[89]
	*Escherichia coli*	[102]
	*Pseudomonas aeruginosa*	[93]
	*Neisseria gonorrhoeae*	[12]
Viral DNA	*Escherichia coli*	[102]
Not specified DNA	*Acholeplasma laidlawii*	[103]
mRNA	*Escherichia coli*	[95]
	*Porphyromonas gingivalis*	[97]
rRNA	*Escherichia coli*	[95]
	*Porphyromonas gingivalis*	[97]
sRNA	*Escherichia coli*	[95]
	*Vibrio cholera*	[90]
	*Clostridium perfringens*	[96]
	*Mycobacterium tuberculosis*	[100]
	*Listeria monocytogenes*	[104]
tRNA	*Escherichia coli*	[95]
Not specified RNA	*Neisseria gonorrhoeae*	[12]

**Table 3:** Protein families identified by proteomic analyses of BMVs.

Proteins	Function	Species	Reference
**Outer membrane porins**			
OmpA and OmpX	Binding to host cell receptors	*Cronobacter sakazakii*	[137]
		*Cronobacter turicensis*	[137]
		*Cronobacter malonaticus*	[137]
OmpA, OmpC, and OmpF	Binding to host cells	*Escherichia coli*	[119]
		*Escherichia coli*△*tolR*	[138]
OmpC	Pore-forming activity	*Salmonella typhi*	[139]
AbOmpA	Binding to host tissue	*Acinetobacter baumannii*	[140]
OprE and OprF	Porin	*Pseudomonas aeruginosa*	[141]
		*Pseudoalteromonas antarctica* NF3	[132]
PorA and PorB	Adherence to host cells	*Neisseria meningitis*	[133]
		*Neisseria meningitis* △*gna33*	[114]
PspA	Binding to human lactoferrin	*Streptococcus pneumoniae*	[142]
**Antibiotic resistance**			
β-lactamase	β-lactamase activity	*Pseudomonas aeruginosa*	[78]
		*Streptococcus pneumoniae*	[108]
		*Moraxella catarrhalis*	[66]
Carbapenemase	Hydrolysis of carbapenem	*Acinetobacter baumannii*	[89]
Cephalosporinases	β-lactamase activity	*Bacteroides* spp.	[107]
Penicillin-binding proteins	Peptidoglycan-based cell wall biogenesis	*Streptococcus pneumoniae*	[123]
TolC	Multidrug efflux pumps	*Escherichia coli*	[67]
		*Escherichia coli*△*tolR*	[138]
Mex	Multidrug efflux pumps	*Pseudomonas aeruginosa*	[143]
		*Pseudoalteromonas antarctica* NF3	[132]
Mtr	Multidrug efflux pumps	*Neisseria meningitis*	[133]
		*Neisseria meningitis*△gna33	[114]
**ABC Transporters**			
BtuB	Vitamin B12 Transporter	*Escherichia coli*	[144]
		*Escherichia coli*△*tolR*	[138]
Tsx	Nucleoside-specific channel-forming protein	*Escherichia coli*	[119]
		*Escherichia coli*△*tolR*	[138]
FecA, FhuA, FhuE, FiuA, FptA	Siderophore transporter	*Neisseria meningitis*△gna33	[114]
		*Escherichia coli*	[144]
		*Clostridium perfringens*	[96]
		*Bacillus subtilis*	[145]
		*Escherichia coli*△*tolR*	[138]
FadL	Long-chain fatty acid transporter	*Escherichia coli*	[119]
		*Escherichia coli*△*tolR*	[138]
Maltoporin LamB	ABC Transporters	*Pseudoalteromonas antarctica* NF3	[119]
		*Escherichia coli*△*tolR*	[138]
		*Escherichia coli*	[144]
ArtI, BraC, FliY, GlnH, HisJ	Amino acid transporter	*Neisseria meningitis*	[133]
Maltose/maltodextrin	Sugar transporter	*Streptococcus pneumoniae*	
Sugar ABC transporter			[142]
**Motility-related proteins**			
Pilus-associated protein	Motility-related proteins	*Neisseria meningitis*	[133]
		*Neisseria meningitis*△gna33	[114]
Flagellin FliC	Motility-related proteins	*Pseudoalteromonas antarctica* NF3	[132]
		*Escherichia coli*	[144]
		*Pseudomonas aeruginosa*	[146]
**Protease/chaperone**			
MSP	Protease	*Legionella pneumophila*	[147]
Protease Pla	Toxicity	*Yersinia pestis*	[79]
Proteases	Enzyme activity	*Streptococcus pneumoniae*	[142]
		*Acinetobacter baumanni*	[148]
		*Yersinia pestis*	[79]
Chaperone SurA	Chaperone	*Pseudoalteromonas antarctica* NF3	[132]
	Chaperone	*Escherichia coli*	[144]
	Chaperone	*Escherichia coli*△*tolR*	[138]
Tail-specific peptidase Prc	Protease	*Escherichia coli*	[144]
	Protease	*Neisseria meningitis*△gna33	[114]
Protease DegQ	Protease	*Pseudoalteromonas antarctica* NF3	[146]
	Protease	*Escherichia coli*△*tolR*	[146]
	Protease	*Escherichia coli*	[144]
**Adhesion/invasion**			
F1 outer fimbrial antigen	Complement binding	*Yersinia pestis*	[79]
Adhesin Ail	Adhesion	*Yersinia pestis*	[79]
UspA1, UspA2	Complement binding	*Moraxella catarrhalis*	[149]
CDT	Toxicity, invasion	*Campylobacter jejuni*	[150]
RgpA, RgpB, Kqp	Host tissue invasion	*Porphyromonas gingivalis*	[151]
Opacity protein	Adhesion and invasion	*Neisseria meningitis*	[133]
OspA, OspB, OspD	Adherence to host cells	*Borrelia burgdorferi*	[152]
IpaB, C, D	Invasion of plasmid antigens	*Shigella* *flexneri*	[153]
Staphopain A	Invasion	*Streptococcus pneumoniae*	[108]
SabA	Adherence	*Helicobacter pylori*	[154]
**Killing of competing bacteria**			
Endopeptidase L5	Peptidoglycan hydrolyse	*Lysobacter* sp.	[131]
*N*-acetylmuramoyl-*L*-alanine amidase	Peptidoglycan hydrolyse	*Streptococcus pneumoniae*	[117]
SLT	Murein hydrolyses	*Neisseria meningitis*	[155]
		*Neisseria meningitis*△gna33	[132]
		*Escherichia coli*△*tolR*	[119]
		*Escherichia coli*	[138]
Mlt	Murein hydrolyse	*Neisseria meningitis*	[155]
		*Pseudoalteromonas antarctica* NF3	[114]
		*Escherichia coli*△*tolR*	[119]
		*Escherichia coli*	[138]
**Host cell modulation**			
α-Hemolysin	Hemolysis	*Pseudomonas aeruginosa*	[153]
		*Pseudoalteromonas antarctica* NF3	[132]
		*Staphylococcus aureus*	[128]
		*Neisseria meningitis*△gna33	[114]
Cytolysin A (ClyA)	Pore-forming ability	*Enterohemorrhagic E. coli*	[125]
		*Salmonella typhi*	[125]
Heat labile enterotoxin (LT)	Toxicity	*Enterotoxigenic E*. *coli*	[129]
Shiga toxin (Stx)	Toxicity	*Shiga toxin producing E*. *coli*	[13]
	Toxicity	*Shigella dysenteriae*	[13]
Cif	Decrease of apical CFTR expression	*Pseudomonas aeruginosa*	[127]
VacA	Vacuolating activity	*Helicobacter pylori*	[154]
Proteolysin	Proteolysis	*Streptococcus pneumoniae*	[156]
β2 toxin	Toxicity	*Streptococcus mutans*	[156]
SEQ, SSaA1, and SSaA2	Evade the host immune system	*Streptococcus pneumoniae*	[123]
Lmo2785	Catalase	*Listeria monocytogenes*	[157]
SOD	Immunomodulatory effect	*Acinetobacter baumannii*	[140]
**Virulence factors**			
Phospholipase C Protease	Hydrolyzes of phospholipids	*Pseudomonas aeruginosa*	[13]
Hcp	Adherence	*Helicobacter pylori*	[154]
Rtx toxin	Cytotoxicity, depolymerizing actin	*Vibrio cholera*	[158]
Macrophage infectivity potentiator (MIP)	Cytotoxicity	*Neisseria meningitis*	[133]
		*Neisseria meningitis*△gna33	[114]
Hemagglutinin	Enzyme activities	*Burkholderia cepacia*	[159]
IgA protease	Protease activity	*Neisseria meningitis*	[133]
		*Pseudoalteromonas antartica* NF3	[132]
InlB and LLO8	Cellular invasion	*Listeria monocytogenes*	[160]
Pertussis toxin (Ptx),	Cytotoxicity	*Bordetella pertussis*	[161]
Adenylate cyclase, hemolysin			
SbI	IgG-binding protein	*Staphylococcus aureus*	[162]
Protective antigen,	Toxicity	*Bacillus anthracis*	[110]
Lethal factor, Edema toxin			
Anthrolysin			
**Cytoplasmic proteins**			
GroEL	60 KDa chaperonin	*Neisseria meningitis*	[133]
		*Escherichia coli*	[144]
ATP-dependent DNA helicase	Interaction	*Staphylococcus aureus*	[123]
EF-Tu	Elongation factor	*Neisseria meningitis*	[133]
		*Staphylococcus aureus*	[123]
		*Clostridium perfringens*	[96]
Pyruvate kinase	Glycolysis	*Staphylococcus aureus*	[123]
Acetate kinase	Phosphorylation	*Staphylococcus aureus*	[123]
Type-3 secretion proteins	Cytoplasmic proteins	*Acinetobacter baumannii*	[140]
Alkaline phosphatase	In vitro enzyme activities	*Pseudomonas aeruginosa*	[143]
DNA gyrase subunit A	Stimulate to antibiotics	*Staphylococcus aureus*	[123]
Hsp60	Heat shock protein	*Legionella pneumophila*	[13]
DnaK	Heat shock 70 kDa protein	*Neisseria meningitis*△gna33	[114]
30S ribosomal protein S1 (RpsA)	Cytoplasmic proteins	*Neisseria meningitis*△gna33	[114]
	Cytoplasmic proteins	*Escherichia coli*	[144]
50S ribosomal protein L7/L12 (RplL)	Cytoplasmic proteins	*Escherichia coli*	[144]
**Coagulation**			
Staphylocoagulase precursor [COL]	coagulation	*Staphylococcus aureus*	[123]
Staphylocoagulase precursor	coagulation	*Staphylococcus aureus*	[123]
Truncated secreted von Willebrand	coagulation	*Staphylococcus aureus*	[123]
Factor-binding protein VWbp	coagulation	*Staphylococcus aureus*	[123]
Others			
Iss	Increased serum survival	*Escherichia coli*	[144]
OstA	Organic solvent tolerance protein	*Pseudoalteromonas antartica* NF3	[132]
	Organic solvent tolerance protein	*Escherichia coli*△*tolR*	[138]
	Organic solvent tolerance protein	*Escherichia coli*	[144]
NADH dehydrogenase-like protein	Oxidation reduction	*Staphylococcus aureus*	[123]
